# The clinical outcomes of combination chemotherapy in elderly patients with advanced biliary tract cancer: an exploratory analysis of JCOG1113

**DOI:** 10.1038/s41598-021-04550-8

**Published:** 2022-01-19

**Authors:** Ikuhiro Yamada, Chigusa Morizane, Takuji Okusaka, Junki Mizusawa, Tomoko Kataoka, Makoto Ueno, Masafumi Ikeda, Naohiro Okano, Akiko Todaka, Satoshi Shimizu, Nobumasa Mizuno, Mitsugu Sekimoto, Kazutoshi Tobimatsu, Hironori Yamaguchi, Tomohiro Nishina, Hirofumi Shirakawa, Yasushi Kojima, Takamasa Oono, Yasuyuki Kawamoto, Masayuki Furukawa, Tomohisa Iwai, Kentaro Sudo, Keiya Okamura, Tatsuya Yamashita, Naoya Kato, Kazuhiko Shioji, Kyouko Shimizu, Toshio Nakagohri, Ken Kamata, Hiroshi Ishii, Junji Furuse, JCOG-HBPOG, Ikuhiro Yamada, Chigusa Morizane, Takuji Okusaka, Junki Mizusawa, Tomoko Kataoka, Makoto Ueno, Masafumi Ikeda, Masato Ozaka, Naohiro Okano, Kazuya Sugimori, Akiko Todaka, Satoshi Shimizu, Nobumasa Mizuno, Mitsugu Sekimoto, Keiji Sano, Kazutoshi Tobimatsu, Akio Katanuma, Kenji Sakai, Hironori Yamaguchi, Tomohiro Nishina, Hirofumi Shirakawa, Yasushi Kojima, Takamasa Oono, Yasuyuki Kawamoto, Masayuki Furukawa, Tomohisa Iwai, Kentaro Sudo, Keiya Okamura, Tatsuya Yamashita, Ichirou Yasuda, Hidenori Takahashi, Naoya Kato, Kazuhiko Shioji, Kyouko Shimizu, Toshio Nakagohri, Ken Kamata, Hiroshi Ishii, Junji Furuse

**Affiliations:** 1 Hepato-Biliary-Pancreatic Medicine Department, Cancer Institute Hospital of Japanese Foundation for Cancer Research, 3-8-31 Ariake, Koto-ku, Tokyo, 135-8550 Japan; 2 Department of Hepatobiliary and Pancreatic Oncology, National Cancer Center Hospital, Chuo-ku, Tokyo, Japan; 3 Japan Clinical Oncology Group Data Center/Operations Office, National Cancer Center Hospital, Chuo-ku, Tokyo, Japan; 4 Department of Gastroenterology, Hepatobiliary and Pancreatic Medical Oncology Division, Kanagawa Cancer Center, Yokohama, Kanagawa, Japan; 5 Department of Hepatobiliary and Pancreatic Oncology, National Cancer Center Hospital East, Kashiwa, Chiba, Japan; 6 Department of Medical Oncology, Kyorin University Faculty of Medicine, Mitaka, Tokyo, Japan; 7 Division of Gastrointestinal Oncology, Shizuoka Cancer Center, Sunto-Gun, Shizuoka, Japan; 8 Department of Gastroenterology, Saitama Cancer Center, Kita-Adachi-Gun, Saitama, Japan; 9 Department of Gastroenterology, Aichi Cancer Center Hospital, Nagoya, Aichi, Japan; 10 Department of Surgery, Kansai Medical University Hospital, Hirakata, Osaka, Japan; 11 Division of Gastroenterology, Department of Internal Medicine, Kobe University Graduate School of Medicine, Kobe, Hyogo, Japan; 12 Department of Surgery, Jichi Medical University, Shimono, Tochigi, Japan; 13 Department of Gastrointestinal Medical Oncology, National Hospital Organization, Shikoku Cancer Center, Matsuyama, Ehime, Japan; 14 Department of Hepato-Biliary-Pancreatic Surgery, Tochigi Cancer Center, Utsunomiya, Tochigi, Japan; 15 Department of Gastroenterology, National Center for Global Health and Medicine, Shinjuku-ku, Tokyo, Japan; 16 Department of Medicine and Bioregulatory Science, Graduate School of Medical Sciences, Kyushu University, Fukuoka, Fukuoka, Japan; 17 Division of Cancer Center, Hokkaido University Hospital, Sapporo, Hokkaido, Japan; 18 Department of Hepato-Biliary-Pancreatology, National Hospital Organization Kyushu Cancer Center, Fukuoka, Fukuoka, Japan; 19 Department of Gastroenterology, Kitasato University School of Medicine, Sagamihara, Kanagawa, Japan; 20 Gastrointestinal Medical Oncology, Chiba Cancer Center, Chiba, Chiba, Japan; 21 Department of Bilio-Pancreatology, Sapporo Kousei General Hospital, Sapporo, Hokkaido, Japan; 22 Department of Gastroenterology, Kanazawa University, Kanazawa, Ishikawa, Japan; 23 Department of Gastroenterology, Graduate School of Medicine, Chiba University, Chiba, Chiba, Japan; 24 Department of Internal Medicine, Niigata Cancer Center Hospital, Niigata, Niigata, Japan; 25 Department of Gastroenterology, Tokyo Women’s Medical University, Shinjuku-ku, Tokyo, Japan; 26 Gastroenterological Surgery, Tokai University School of Medicine, Isehara, Kanagawa, Japan; 27 Department of Gastroenterology and Hepatology, Kindai University Faculty of Medicine, Osakasayama, Osaka, Japan; 28 Gastroenterological Center, Yokohama City University Medical Center, Yokohama, Kanagawa, Japan; 29 Department of Surgery, Teikyo University School of Medicine, Itabashi-ku, Tokyo, Japan; 30 Center for Gastroenterology, Teine Keijinkai Hospital, Sapporo, Hokkaido, Japan; 31 Department of Hepatobiliary and Pancreatic Surgery, Osaka National Hospital, Chuo-ku, Osaka, Japan; 32 Department of Gastroenterology and Hematology, Faculty of Medicine, University of Toyama, Toyama, Toyama, Japan; 33 Department of Gastroenterological Surgery, Osaka International Cancer Institute, Chuo-ku, Osaka, Japan

**Keywords:** Gastroenterology, Oncology

## Abstract

In the FUGA-BT trial (JCOG1113), gemcitabine plus S-1 (GS) showed non-inferiority to gemcitabine plus cisplatin (GC) in overall survival (OS) with good tolerance for patients with advanced biliary tract cancer (BTC). We performed a subgroup analysis focused on the elderly cohort of this trial. All 354 enrolled patients in JCOG1113 were classify into two groups; < 75 (non-elderly) and ≥ 75 years (elderly) group. We investigated the influence of age on the safety analysis, including the incidence of chemotherapeutic adverse events and the efficacy analysis, including OS. There were no remarkable differences in OS between the elderly (*n* = 60) and the non-elderly groups (*n* = 294). In the elderly group, median OS was 12.7 and 17.7 months for those who received GC (*n* = 20) and GS (*n* = 40), respectively. The prevalence of all-grade adverse events was similar between the elderly and the non-elderly groups. However, among the elderly group, Grade ≥ 3 hematological adverse events were more frequently observed in the GC arm than in the GS arm. The clinical outcomes of combination chemotherapy in elderly patients with advanced BTC were comparable to non-elderly patients. GS may be the more favorable treatment for elderly patients with advanced BTC.

## Introduction

Biliary tract cancer (BTC) is a malignant tumor arising from the biliary tract, which includes the intrahepatic bile duct, extrahepatic bile duct, gallbladder (GB), and ampulla of Vater. The number of patients with BTC has been increasing in Japan, and 63.9% of patients are over 75 years^1,2^. Most patients have been diagnosed with locally advanced, metastatic, or recurrent disease, therefore palliative chemotherapy is important for improving the prognosis of patients with BTC. However, the 5-year overall survival remains dismal at 10–20%^3,4^.

With an aging global population, there is a growing need to evaluate treatment outcomes in elderly patients with cancer. Several studies have reported the clinical outcomes of palliative chemotherapy in elderly patients with BTC^5,6^. Although gemcitabine plus cisplatin combination therapy (GC) is currently the standard regimen following the ABC-02 trial^7^, physicians in general clinical practice may be hesitant to administer GC for elderly patients due to perceptions of the potential for increased toxicity in a population with a high proportion of comorbidity.

Recently, Hepatobiliary and Pancreatic Oncology Group of Japan Clinical Oncology Group (JCOG) reported the results of the randomized phase III trial (FUGA-BT/JCOG1113) that compared GC and gemcitabine plus S-1 (GS), an oral fluoropyrimidine combination consisting of tegafur (a prodrug of 5-fluorouracil [5-FU]) and the 5-FU modulators gimeracil and oteracil, combination therapy as a first-line treatment for patients with advanced BTC^8^. In JCOG1113, GS showed non-inferiority to GC in overall survival (OS) with good tolerance (median OS: 13.4 months with GC and 15.1 months with GS, hazard ratio 0.945; 90% confidence interval 0.78–1.15).

For elderly patients, GS may also show good tolerance, however, there is less data regarding clinical outcomes of combination chemotherapy in elderly patients with advanced BTC. Therefore, we planned the present subgroup analysis focused on the elderly cohort of JCOG1113.

## Methods

### Study design and patients

JCOG1113 was a randomized phase III trial that enrolled patients from 33 institutions in Japan^8^. Main eligibility criteria were: histologically proven adenocarcinoma or adenosquamous carcinoma of the biliary tract; unresectable or recurrent disease; no previous chemotherapy; Eastern Cooperative Oncology Group performance status of 0 or 1; preserved major organ function; and written informed consent to participate. The study protocol was approved by the institutional review board of each participating institution (Cancer Institute Hospital of Japanese Foundation for Cancer Research, National Cancer Center Hospital, Kanagawa Cancer Center, National Cancer Center Hospital East, Kyorin University Faculty of Medicine, Yokohama City University Medical Center, Shizuoka Cancer Center, Saitama Cancer Center, Aichi Cancer Center Hospital, Kansai Medical University Hospital, Teikyo University School of Medicine, Kobe University Graduate School of Medicine, Teine Keijinkai Hospital, Osaka National Hospital, Jichi Medical University, National Hospital Organization Shikoku Cancer Center, Tochigi Cancer Center, National Center for Global Health and Medicine, Kyushu University, Hokkaido University Hospital, National Hospital Organization Kyushu Cancer Center, Kitasato University School of Medicine, Chiba Cancer Center, Sapporo Kousei General Hospital, Kanazawa University, University of Toyama, Osaka International Cancer Institute, Chiba University, Niigata Cancer Center Hospital, Tokyo Women's Medical University, Tokai university School of Medicine, Kindai University Faculty of Medicine). The study was conducted in accordance with the ethical standards established in the 1964 Declaration of Helsinki and its later amendments.

Between May 8, 2013 and March 4, 2016, a total of 354 patients were enrolled to JCOG1113; 175 were assigned to the GC arm and 179 to the GS arm. For this subgroup analysis to investigate the influence of age, all registered patients of JCOG1113 were stratified by age; < 75 (non-elderly group) and ≥ 75 years (elderly group). Due to the growing of global aging society and most patients with BTC are over 75 years in Japan, the age 75 years was selected that it was an acceptable cut-off value to define the ‘elderly’ population. Additionally, the efficacy and the safety data were compared by regimen (GC vs. GS) in each group.

### Outcomes

The primary endpoint of this subgroup analysis was OS. The secondary endpoints were progression-free survival (PFS), response rate (RR), adverse events (AEs), serious AEs, clinically significant AEs, and percent planned dose administered. Clinically significant AEs were predefined as those of Grade ≥ 2, including fatigue, anorexia, nausea, vomiting, oral mucositis, and diarrhea, occurring at least once during the monitoring period, which was the duration from the start of treatment until completion of eight cycles of treatment, or 24 weeks, whichever was longer. OS was calculated from the date of randomization to the date of death, or censored on the date of last contact for surviving patients. PFS was counted from the date on which disease progression or death was detected, or was censored on the last date when progression-free status was confirmed. Tumor response was assessed every six weeks according to the Response Evaluation Criteria in Solid Tumors version 1.1. RR was calculated without confirmation.

### Treatment

Patients were randomly assigned to either the GC or GS treatment arm. Patients assigned to GC arm received gemcitabine (1000 mg/m^2^) and cisplatin (25 mg/m^2^) via infusion on days 1 and 8; this regimen was repeated every 3 weeks. Cisplatin was administered up to 16 times (total 400 mg/m^2^) unless patients met the termination criteria. After cisplatin termination, patients received gemcitabine (1000 mg/m^2^) via infusion on days 1, 8, and 15, repeated every 4 weeks. Patients assigned to GS arm received gemcitabine (1000 mg/m^2^) via infusion on days 1 and 8. S-1 was administered orally twice daily (60 mg/day for a body surface area [BSA] < 1.25 m^2^, 80 mg/day for a BSA between 1.25 and < 1.50 m^2^, and 100 mg/day for a BSA ≥ 1.50 m^2^) on days 1–14. This regimen was repeated every 3 weeks.

### Statistical analysis

OS and PFS were estimated by the Kaplan–Meier method. Hazard ratios and corresponding 95% confidence intervals [CIs] were estimated using the Cox regression hazard model. CIs of RR and clinically significant AEs were calculated by Clopper-Pearson method. Statistical analyses were performed with SAS version 9.4. All statistical analyses were conducted at the JCOG Data Center.

## Results

### Patient characteristics

**Figure 1:**
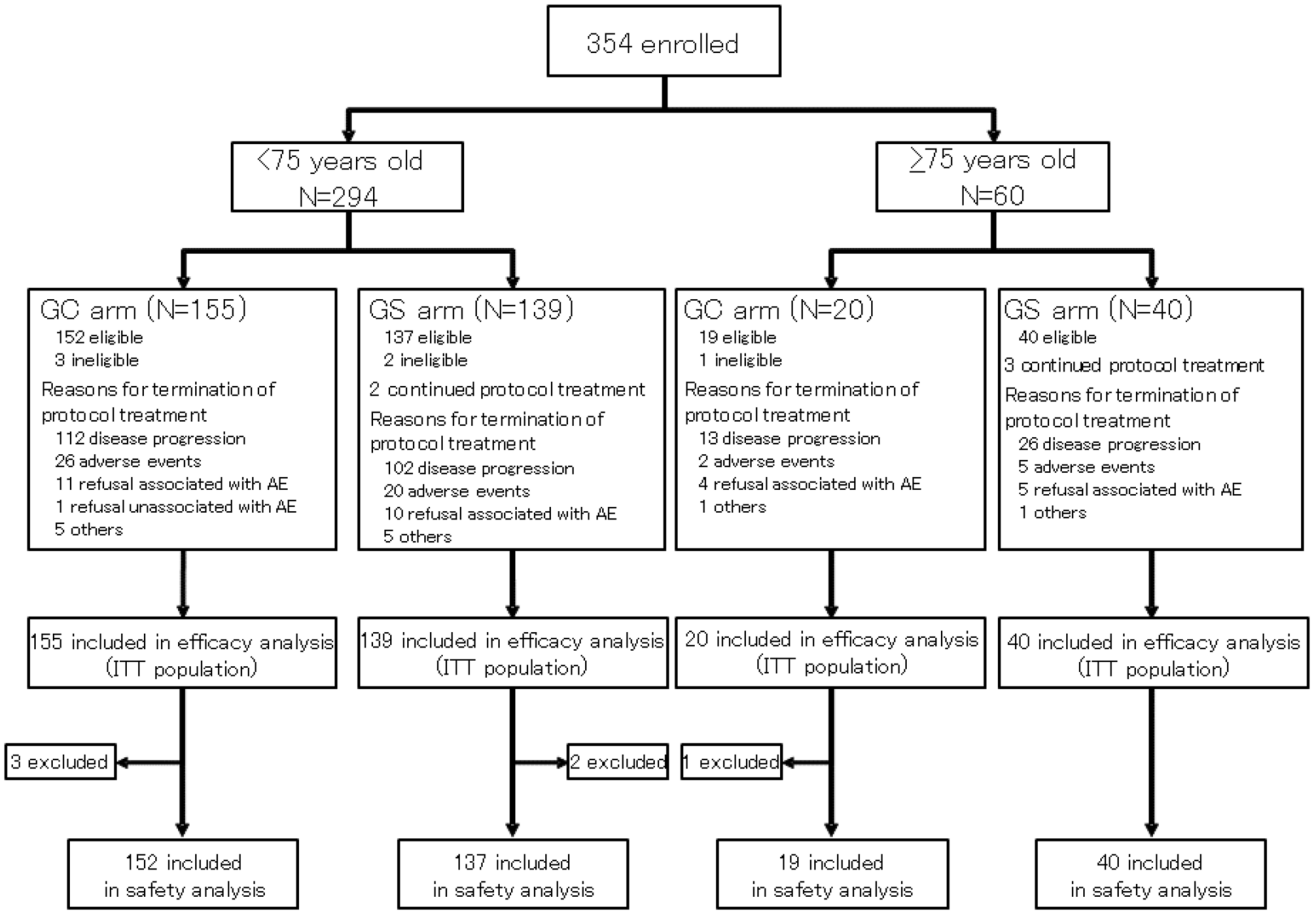
Patients flow diagram.

Of all registered 354 patients in JCOG1113, 60 patients aged ≥ 75 years were classified into the elderly group (20 in GC arm and 40 in GS arm), and 294 patients aged < 75 years were classified into the non-elderly group (155 in GC arm and 139 in GS arm). One patient in the GC arm was determined to be ineligible after registration; the patient was included in the efficacy analysis but excluded from the safety analysis (Fig. 1). Patient baseline characteristics were similar between the two groups (Table 1), and they were also similar between the treatment arms (GC arm and GS arm) in the elderly group.

**Table 1:** Baseline characteristics (intention-to-treat population).

		< 75 years old				≥ 75 years old			
		GC (*n* = 155)		GS (*n* = 139)		GC (*n* = 20)		GS (*n* = 40)	
Age	Median (range)	66	(41–74)	65	(27–74)	76	(75–78)	76	(75–79)
Sex	Male	91	(58.7%)	75	(54.0%)	8	(40.0%)	22	(55.0%)
	Female	64	(41.3%)	64	(46.0%)	12	(60.0%)	18	(45.0%)
ECOG PS	0	117	(75.5%)	97	(69.8%)	13	(65.0%)	27	(67.5%)
	1	38	(24.5%)	42	(30.2%)	7	(35.0%)	13	(32.5%)
Primary site	Gallbladder	58	(37.4%)	56	(40.3%)	10	(50.0%)	13	(32.5%)
	Intrahepatic bile duct	47	(30.3%)	38	(27.3%)	3	(15.0%)	6	(15.0%)
	Extrahepatic bile duct	43	(27.7%)	41	(29.5%)	6	(30.0%)	18	(45.0%)
	-Perihilar	27	(17.4%)	21	(15.1%)	2	(10.0%)	11	(27.5%)
	-Distal	16	(10.3%)	20	(14.4%)	4	(20.0%)	7	(17.5%)
	Ampulla of Vater	6	(3.9%)	3	(2.2%)	1	(5.0%)	3	(7.5%)
	Other (ineligible)	1	(0.6%)	1	(0.7%)	0		0	
Biliary drainage	Present	61	(39.4%)	61	(43.9%)	11	(55.0%)	21	(52.5%)
Resection	Present	48	(31.0%)	45	(32.4%)	3	(15.0%)	9	(22.5%)
Stage	Locally advanced	29	(18.7%)	24	(17.3%)	2	(10.0%)	8	(20.0%)
	Metastatic	91	(58.7%)	82	(59.0%)	15	(75.0%)	25	(62.5%)
	Recurrence	34	(21.9%)	32	(23.0%)	3	(15.0%)	7	(17.5%)
	Missing (ineligible)	1	(0.6%)	1	(0.7%)				

ECOG PS, Eastern Cooperative Oncology Group performance status; GC, gemcitabine plus cisplatin; GS, gemcitabine plus S-1.

### Treatment compliance

Patient compliance with each treatment is shown in Table 2. There were no remarkable differences in dose reduction or percent planned dose between elderly and non-elderly patients within treatment arms. In elderly patients, those metrics were also similar between the treatment arms. Reasons for the termination of protocol treatment were similar between elderly and non-elderly patients (proportion of disease progression; 32.2% in elderly patients and 38.4% in non-elderly patients; incidence of AEs or patient’s refusal associated with AEs; 16.9% in elderly patients and 14.9% in non-elderly patients). In elderly patients, those were similar between treatment arms (proportion of disease progression; 36.8% in the GC arm and 30.0% in the GS arm; incidence of AEs or patient’s refusal associated with AEs, 15.8% in the GC arm and 17.5% in the GS arm).

**Table 2:** Treatment compliance according to age in GC and GS.

		< 75 years old		≥ 75 years old	
		GC (*n* = 152)	GS (*n* = 137)	GC (*n* = 19)	GS (*n* = 40)
Treatment cycles	Median	8	8	8	8.5
	Range	1–36	1–63	2–40	1–95
Dose reduction	Gemcitabine (%)	39 (25.7%)	38 (27.7%)	5 (26.3%)	13 (32.5%)
	S-1 (%)		29 (21.2%)		9 (22.5%)
Median % planned dose	Gemcitabine	75.0%	80.0%	75.0%	86.6%
	Cisplatin	75.0%		77.8%	
	S-1		75.7%		82.1%

GC, gemcitabine plus cisplatin; GS, gemcitabine plus S-1.

### Adverse events

The prevalence of all-grade AEs and clinically significant AEs were similar between elderly and non-elderly patients. In 59 elderly patients (19 in GC arm and 40 in GS arm), Grade ≥ 3 hematological adverse events were more frequent in the GC arm compared to the GS arm. Considering all-Grade non-hematological adverse events, nausea (26.3%), and vomiting (15.8%) were more frequent in the GC arm, while anorexia (40.0%), fever (35.0%), oral mucositis (25.0%) and diarrhea (22.5%) were more frequent in the GS arm (Table 3). Clinically significant AEs were observed in 25.0% and 32.5% and Grade ≥ 3 clinically significant AEs were observed in 15.0% and 10.0% of elderly patients in the GC and the GS arms, respectively.

**Table 3:** Summary of adverse events data according to CTCAE v4.0 (safety analysis set).

	< 75 years old				≥ 75 years old			
	GC (*n* = 152)		GS (*n* = 137)		GC (*n* = 19)		GS (*n* = 40)	
	All-grade (%)	Gr.3–4 (%)	All-grade (%)	Gr.3–4 (%)	All-grade (%)	Gr.3–4 (%)	All-grade (%)	Gr.3–4 (%)
Decreased white blood cell count	77.0	29.6	78.1	24.8	94.7	47.4	75.0	25.0
Anemia	98.0	21.1	97.8	5.1	100	47.4	100	10.0
Decreased platelet count	81.6	14.5	75.2	7.3	89.5	31.6	85.0	7.5
Decreased neutrophil count	84.9	59.2	86.1	60.6	100	73.7	87.5	57.5
Diarrhea	14.5	1.3	20.4	1.5	5.3	0	22.5	0
Oral mucositis	14.5	0	29.9	0	0	0	25.0	7.5
Palmar-plantar erythrodysesthesia syndrome	0.7	0	5.8	0.7	0	0	0	0
Rash maculopapular	7.9	0	24.1	5.1	21.1	0	22.5	10.0
Biliary tract infection	18.4	18.4	19.7	19.7	26.3	26.3	25.0	25.0
Fatigue	51.3	3.3	43.8	5.8	47.4	15.8	45.0	5.0
Fever	30.9	0.7	29.9	2.9	15.8	5.3	35.0	0
Nausea	38.2	0.7	35.0	1.5	26.3	0	20.0	2.5
Vomiting	11.8	0.7	10.9	0.7	15.8	0	10.0	0
Pneumonitis	1.3	0.7	1.5	0	0	0	5.0	0
Anorexia	42.8	5.3	39.4	5.8	26.3	10.5	40.0	5.0
Febrile neutropenia	2.0	2.0	2.2	2.2	5.3	5.3	0	0
Peripheral sensory neuropathy	17.1	0	3.6	0	5.3	0	2.5	0

There were three treatment-related deaths in the GC group and none in the GS group. GC, gemcitabine plus cisplatin; GS, gemcitabine plus S-1.

In JCOG1113, three treatment-related deaths occurred in the GC arm, while none occurred in the GS arm. Of these deaths, one (disseminated intravascular coagulation from cholangitis and/or liver abscess) was an elderly patient.

### Efficacy

**Figure 2:**
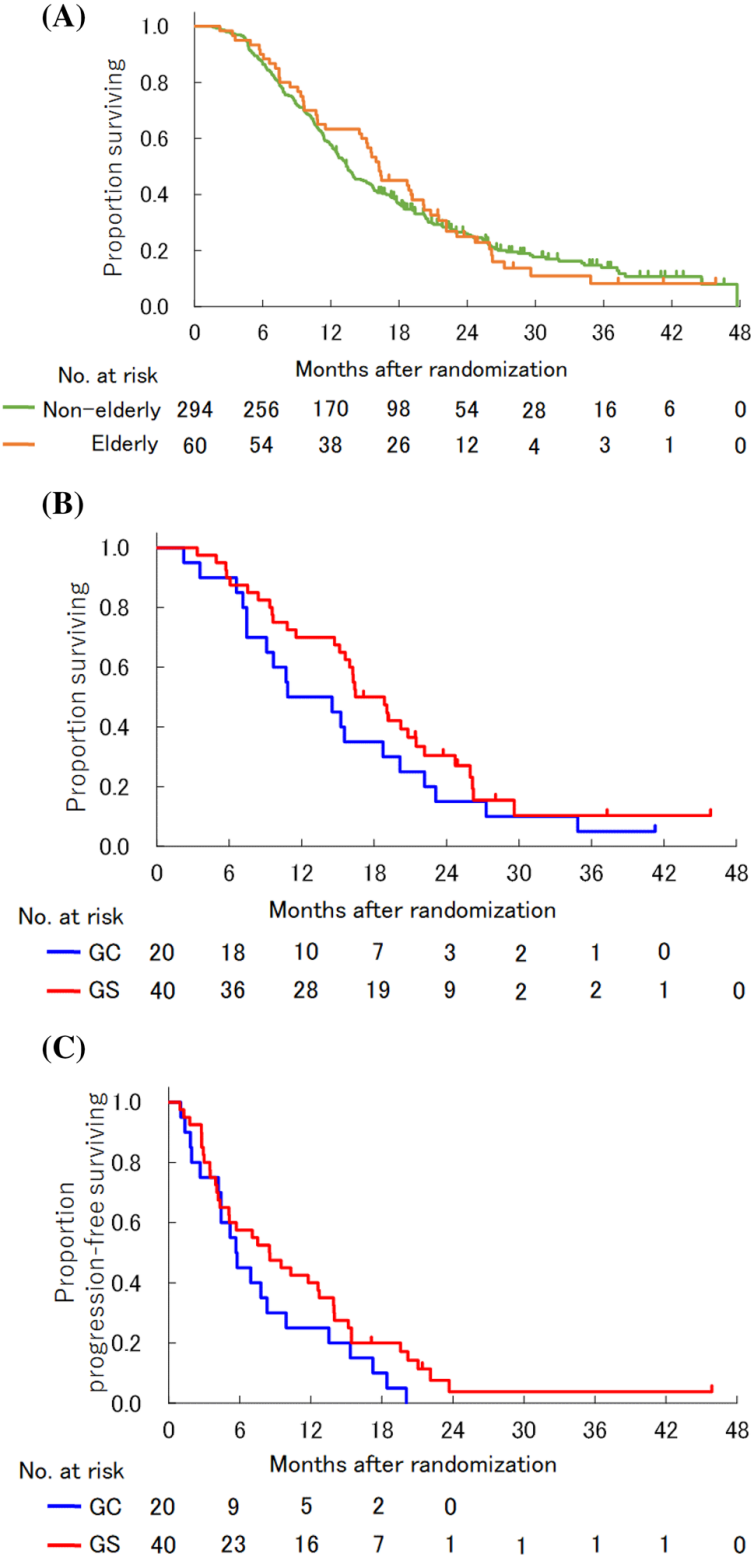
Overall survival and progression-free survival (intention-to-treat population). (**A**) Overall survival for elderly and non-elderly patients. (**B**) Overall survival for elderly patients in the GC and GS group. (**C**) Progression-free survival for elderly patients in the GC and GS group. Vertical lines on curves indicate patients censored on the date of their last follow-up. GC, gemcitabine plus cisplatin; GS, gemcitabine plus S-1.

The median OS of the elderly patients was 16.2 (95% CI 11.5–20.1) months, whereas that of the non-elderly patients was 13.4 (95% CI 12.4–15.2) months (HR 0.959, 95% CI 0.709–1.299). There were no remarkable differences in OS between elderly patients and non-elderly patients. In elderly patients, the median OS in the GC arm was 12.7 (95% CI 7.5–20.1) months versus 17.7 (95% CI 15.1–21.5) months in the GS arm (HR 0.693, 95% CI 0.391–1.227). The median PFS was 5.7 (95% CI 2.7–9.9) months in the GC arm and 8.5 (95% CI 4.3–12.7) months in GS arm (HR 0.650, 95% CI 0.371–1.137) (Fig. 2). Among elderly patients with measurable lesions, the RRs were 50.0% (8/16; 95% CI 24.7%–75.4%) in the GC arm and 30.0% (9/30; 95% CI 14.7%–49.4%) in the GS arm. There were no remarkable differences in OS, PFS, or RR between treatment arms in elderly patients.

### Second-line treatment

A total of 46 (78.0%) elderly patients received second-line treatment. In the GC arm, 13 patients (68.4%) received second-line chemotherapy, mainly S-1 monotherapy (8 patients). In the GS arm, 33 patients (82.5%) received second-line chemotherapy, mainly GC (20 patients; Table 4).

**Table 4:** Details of second-line treatment in GC and GS.

		< 75 years old		≥ 75 years old	
		GC (*n* = 152)	GS (*n* = 137)	GC (*n* = 19)	GS (*n* = 40)
Received second-line treatment *n* (%)		130 (85.5)	110 (80.3)	13 (68.4)	33 (82.5)
Regimens	GC	6	77	0	20
	GS	16	4	1	0
	Gemcitabine monotherapy	14	8	2	6
	S-1 monotherapy	68	9	8	4
	Others	26	12	2	3

GC, gemcitabine plus cisplatin; GS, gemcitabine plus S-1.

## Discussion

This subgroup analysis indicates that survival benefits with gemcitabine-based combination chemotherapy were similar between elderly and non-elderly patients. Furthermore, the frequency of all-Grade AEs was also similar between elderly and non-elderly patients. Our present results suggest that elderly patients with good general condition are expected to achieve clinical outcomes with the combination chemotherapy comparable to non-elderly patients treated for BTC. In elderly patients, OS and PFS of GS arm was tended to be better than that of GC arm, and Grade ≥ 3 hematological adverse events were observed more frequently in GC arm.

Several studies have investigated the difference of survival benefits by age in patients with BTC who received palliative chemotherapy. McNamara et al. reported the results of meta-analysis of 13 trials of systemic therapy for BTC, including 1163 patients^6^. The meta-analysis included 260 patients (22%) ≥ 70 years of age and the primary analysis demonstrated that PFS for those < 70 and ≥ 70 years was 6.0 and 5.0 months, and OS was 10.2 and 8.8 months, respectively. Their multivariable analysis of those data demonstrated that age was not associated with either PFS or OS. Similarly to our present study, they concluded that survival in elderly patients treated with combination chemotherapy is similar to that of non-elderly patients. Additionally, Horgan et al. reported results from a large retrospective study of elderly patients with BTC that included 913 patients^9^. The study included 321 patients ≥ 70 years of age and they concluded that active therapy is associated with similar survival benefit regardless of age. While some retrospective studies showed similar results^10,11^, other studies showed discrepant results that elderly patients treated with palliative chemotherapy for BTC had a poorer prognosis than non-elderly patients^12^. Only "fit" elderly patients are enrolled to clinical trials, however, many elderly patients are not fit in the real world. Therefore, indication of aggressive treatments for elderly patients has remained controversial and selection bias may have influenced these discrepant results. In these previous studies, the age 70 years was selected as a cut-off value to define the ‘elderly’ population. However, now that the global population is aging, the age of patients with BTC is also increasing, especially in Japan, a lot of patients with BTC are over 75 years old^2^. There is an increasing need to develop standard treatment for patients at an older age than before. Therefore, in this exploratory analysis, we adopted 75 years as a cut-off value, an older age than the age selected in the previous studies.

Elderly patients with cancer are a heterogeneous population. Aggressive treatment may be difficult for some elderly patients to tolerate; however, many fit elderly patients may not be adequately treated based only on their chronological age. Thus, in recent years, a comprehensive assessment of elderly patients is considered to be important to promote more individualized therapeutic approaches in geriatric oncology. A comprehensive geriatric assessment (CGA) is a multidisciplinary evaluation of an elderly person, not only regarding physical status, but also functional, cognitive, and psychosocial status^13^. Several studies have shown that CGA can identify patients at an increased risk for mortality and be useful in making decisions concerning appropriate treatment for elderly patients with cancer^14–16^. Similarly, decisions regarding treatment of elderly patients for BTC should not be dictated by chronological age alone, and multidisciplinary assessment should be considered to determine which patients may benefit from combination chemotherapy.

Notably, elderly patients treated with GS had a trend towards improved OS and PFS compared with those treated with GC in the present study. In terms of toxicities, Grade ≥ 3 hematological adverse events were observed more frequently in elderly patients treated with GC than in those treated with GS. In consideration of the above, GS is more suitable for elderly patients with low hematopoietic function levels at baseline. Additionally, GC requires hydration to reduce renal toxicity; however, elderly patients with cardiac and/or renal disease are unsuitable to receive large volume fluid infusions. Therefore, GS is more convenient for elderly patients as it does not require hydration, thereby reducing in-hospital time. However, the specific mechanisms leading to favorable outcomes with GS in elderly patients remains unclear and further study is needed. On the other hand, predefined clinically significant AEs such as anorexia, oral mucositis, nausea and vomiting were observed more frequently in elderly patients treated with GS than in those treated with GC. Elderly patients are more likely to suffer from dehydration due to poor oral intake, therefore it may be better not to administer GS to elderly patients with unstable oral intake. Imaoka et al. analyzed the clinical outcomes of elderly patients treated with gemcitabine alone, S-1 alone, or GS in prospective trials enrolling patients with unresectable pancreatic cancer that included 90 patients ≥ 70 years of age treated with GS^17^. There were three treatment-related deaths (interstitial lung disease, cerebrovascular disorder and an unknown cause associated with myelosuppression) in elderly patients treated with GS and two of the three patients were ≥ 80 years. There are only few reports of GS treatment for elderly patients and elderly candidates for GS therapy should be carefully selected.

One limitation of our study was the lack of international standardization, as all registered patients were from Japanese institutions. More severe gastrointestinal toxicities associated with S-1 have been reported among patients outside Asia^18,19^. Thus, these results should be considered carefully if GS is used to treat Western patients. Another limitation was the lack of quality of life (QoL) assessment. Maintaining QoL is a key goal of therapy and it affects the treatment decisions of many patients. Bridgewater et al. reported that the survival advantage of GC compared to gemcitabine alone was not associated with an improvement or deterioration of the QoL in the ABC-02 trial^20^. In the advanced disease setting, we need to discuss treatment goals with elderly patients, as QoL has been shown to be of greater importance than survival among elderly patients^21^. Additionally, our analysis was limited by the inclusion of a small number of elderly patients from 75 to 79 years old and lack of adequate statistical power. Therefore, detailed subgroup analysis was not meaningful and favorable outcomes associated with GS in elderly patients should be interpreted with caution.

In conclusion, the clinical outcomes of combination chemotherapy in elderly patients with advanced BTC were comparable to those in non-elderly patients. From the view point of the trend toward better prognosis, less hematological toxicities, and unnecessariness of hydration, GS may be a more favorable treatment than GC for elderly patients with advanced BTC.
